# Use of Time-Dependent Multispectral Representation of Magnetic Barkhausen Noise Signals for the Needs of Non-Destructive Evaluation of Steel Materials

**DOI:** 10.3390/s19061443

**Published:** 2019-03-24

**Authors:** Michal Maciusowicz, Grzegorz Psuj

**Affiliations:** Department of Electrical and Computer Engineering, Faculty of Electrical Engineering, West Pomeranian University of Technology, Szczecin, al. Piastow 17, 70-310 Szczecin, Poland

**Keywords:** Barkhausen noise, feature extraction, magnetization process, multidimensional signal processing, non-destructive testing, spectral analysis, spectrogram, STFT, stress evaluation

## Abstract

Due to the existing relationship between microstructural properties and magnetic ones of the ferromagnetic materials, the application potential of the magnetic Barkhausen noise (BN) method to non-destructive testing is constantly growing. However, the stochastic nature of the Barkhausen effect requires the use of advanced signal processing methods. Recently, the need to apply time-frequency (*TF*) transformations to the processing of BN signals arose. However, various *TF* methods have been used in the majority of cases for qualitative signal conditioning and no extensive analysis of *TF*-based information has been conducted so far. Therefore, in this paper, the wide analysis of BN *TF* representation was carried out. Considering the properties of *TF* transformations, the Short-Time Fourier Transform (STFT) was used. A procedure for definition of the envelopes of the *TF* characteristic was proposed. To verify the quality of extracted features, an analysis was performed on the basis of BN signals acquired during stress loading experiments of steel elements. First, the preliminary experiments were processed for various parameters of the measuring system and calculation procedures. The feature extraction procedure was performed for different modes of *TF* representations. Finally, the distributions of *TF* features over the loading stages are presented and their information content was validated using commonly used features derived from time *T* and frequency *F* domains.

## 1. Introduction

Ferromagnetic materials microstructure is defined by regions known as magnetic domains. The domains are micro-areas of spontaneous, homogeneous magnetization, separated from each other by thin layers of magnetic discontinuity of atoms moments called Bloch walls. The direction of magnetization between the two domains is gradually changed by the rotation of the resulting magnetic moment [1,2,3]. It is well known that the magnetic structure depends on the mechanical one [4,5,6,7,8,9]. Thus, by observation of magnetization process under the alternating field conditions, it is possible to monitor the changes of a material’s state. The relationship is reflected in parameters such as magnetic coercivity, remanence or initial magnetic permeability and magnetic hysteresis loop loss [5,6,7,10,11,12]. The areas that have non-homogeneities (i.e., of micro-stress concentration or dislocations) do not allow the Bloch walls to rotate freely and influences the reorganization of the domain structure during the magnetization process. Consequently, these obstacles arising in the material require additional energy and cause discontinuous changes of the magnetic state. This phenomenon has been studied and gradually used for non-destructive testing (NDT) of materials [4,5,6,11,12,13,14,15,16,17,18,19,20,21,22,23,24,25,26,27,28,29,30,31,32,33]. The stepwise change of the magnetization level during operation of the alternating magnetizing field can be observed by coil placed in the neighbourhood of the material. As a result, a voltage *U*_BN_ called the Barkhausen Noise (BN) is induced in coil. The Magnetic Barkhausen Noise method (MBN) is used mainly for [29]: detection and evaluation of fatigue and stress [4,5,6,11,12,16,17,22,24,26,31,32,34,35,36,37,38,39], material microstructure [19,20,28,37,38,40] and microhardness (i.e., hardening level, case depth) analysis [13,14,23,27].

There are two major aspects that have to be considered during the application of the MBN method: the construction of the BN sensing unit and the signal processing procedure. The first one, in case of NDT, is generally consistent. Most frequently, the transducers used for BN observation are consisting of two sections, magnetization and pick-up. The material is magnetized by the yoke while the signal is collected by sensing elements (e.g., pancake coil) placed between the pole pieces [32]. However, the second aspect is an important factor which may significantly influence the overall effectiveness of the method. This demonstrates the need for development and implementation of data mining procedures. Therefore, in this paper a work allowing obtaining new characteristics from time-frequency *TF* domain of BN signal was carried out.

**Figure 1:**
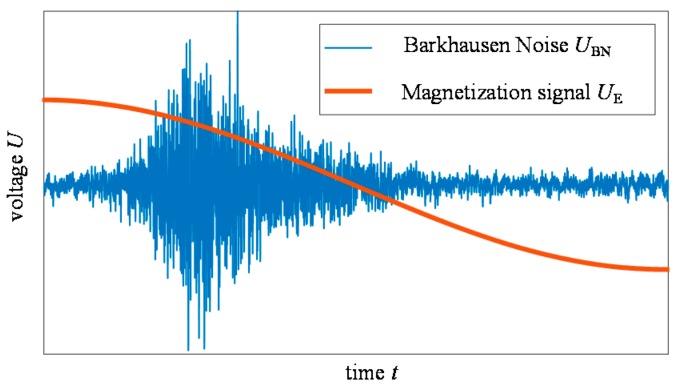
View of the exemplary Barkhausen noise and magnetization signals.

The BN has a stochastic nature and therefore it requires the application of advanced signals processing methods allowing extraction of signal features containing critical information about the state of the material. The exemplary BN signal is presented in Figure 1. Many factors defining material state may affect various properties of BN signals. Mostly the analysis of BN is carried out in time *T* domain [4,6,11,12,19,20,34,35,36,37,38]; however the frequency *F* characteristic is also considered, especially for evaluation of material properties [12,13,14,26,27,28,39]. In case of *T* representation, the BN signal can be analysed by calculating parameters directly from the raw signal. One of the most commonly used parameters in the *T* domain is the root-mean-square (RMS) value of the BN signal [4,11,12,18,33]. Many other parameters are also calculated directly from raw BN signal such as energy, standard deviation, variance or number of impulses. Furthermore, in order to extend the range of features being extracted, frequently the envelope of BN signal is also used. Thus, the shape of the envelope can be additionally studied to complement the information by the analysis of features such as peak height, peak position, full-width-at-half module, skewness or kurtosis [11,12,19,24,25]. As the dynamics of BN signal can also vary, the *F* domain has been also used. It was presented that by using a quantitative approach to *F* domain analysis, one can also evaluate the applied and residual stress or hardness level value [14,26,27,28]. For example, it has also been shown that the value of power spectral density carries other information about changes in the material than the RMS value of MBN. However, none of the parameters fully describes all aspects of physical impact on the ferromagnetic material as it was discussed in [11,24] and the relationship between the magnetic properties and the mechanical ones still requires constant and further study. The signal representation in *T* as well as in *F* domain changes simultaneously. Moreover, the dynamics of the phenomenon depends on many factors, including the stress state of the structure [7,13,19,20,25,29,33]. In such cases, the traditional methods of frequency analysis are not adequate while they generalize the dynamics of non-stationary signal over whole considered period of time. Thus, the combined *TF* domain analysis can provide one with complementary information and should also be carried out [12,15,16,21,22,28,30,41,42].

There are several works presenting the possibility of using the *TF* transformation for observation of e.g., stress, fatigue or hardness influence on MBN. However, they mainly refer to qualitative evaluation of material conditions and there was only little work done on extended quantitative extraction of information from obtained *TF* representations. In [28] *TF* representation was used for qualitative assessment of the time-frequency distributions and observation of frequency bandwidth at low and high levels of magnetizing field. The application of *TF* transformation to signal conditioning and reconstruction for the needs of stress and hardness evaluation was presented in [30]. The BN signal was decomposed into the *TF* domain where it was modified and then reconstructed resulting in reduction of background signal level. *TF* representation was also used for qualitative assessment of measuring conditions of continuous BN signal under applied stress [17], while in [21] various transformation methods were used to observe the influence of the hardness level on signal expressed in *TF* domain and theirs comparison was presented. In the second case the area of iso-level curve, cut at particular magnitude, was used to quantify the material changes. In [15,16] transformation coefficients were used for assessment of residual stress level and fatigue crack evaluation. Nevertheless, as the *TF* characteristics are complicated in shape and there are many aspects influencing the BN signal properties, a detailed analysis and quantitative description should be carried out. It must be underlined that the analysis must consider various aspects of the *TF* characteristics changes such as symmetry, shift of the centre in *T*- or *F*-axis, flatness, uniformity or monotonicity, etc. While the *TF* representation is preserving important information, using only a few parameters, such as peak value or peak position, it does not allow extracting whole information content [22]. Therefore, the aim of this paper was to evaluate the information content of the *TF* representation of BN signal in the context of non-destructive testing of material changes using broad feature vector. Thus, in this paper the wide analysis of BN *TF* representation was carried out. The procedure for the description of *TF* characteristics by two envelopes over the *T*- and *F*-axis was proposed and conducted. The general processing methods used for non-stationary signals characterization were also applied. It resulted in a significant number of features allowing quantitative description of various aspects of changes in obtained transformations distributions.

The second part, which was also considered before conducting the work, was the transformation type. There are various transformation methods used under two major strategies of *TF* space division, defined in accordance to a consistency of sliding window size, and thus allowing building of two types of representations: time-scale *TS* (i.e., wavelet) [15,16,21,23] or time-frequency *TF* [17,21,22,43]. The first one is characterized by variable resolution of time-frequency spans and results in obtaining representation in the form of scalograms. The second way to decompose the signal is to apply the constant resolution of the time-frequency spans, which results in achieving representation in the form of spectrograms. Examples of the first type of decomposition are frequently used various types of wavelet transform WT as these allows good fitting rate to signals with stochastic characteristic. The WT founds its application to residual stress, fatigue crack or hardening evaluation [15,16,21,23]. However, these methods, resulting in non-uniform resolution of *TF* representation, do not allow the systematic comparison of frequency bandwidth in equal successive spans of the magnetization period under different material conditions [21]. In accordance, application of constant resolution in the division of the time-frequency space allows observation of changes in the dynamics of the signal for subsequent magnetizing temporal moments. This may have a significant impact on the efficiency of the whole non-destructive evaluation procedure along with interpretation of results. Consequently, it can be especially beneficial when analysing changes or variations in material properties, at different depths in the object under test. One of the simplest to implement transformation method of this type is short-time Fourier transform (STFT). Even though it presents some limitations in terms of frequency resolution, it is easy to use (has relatively low computational requirements) and provide similar information content of BN signal as more advanced transformations [21]. This is crucial factor in many industrial applications. Therefore, considering all those aspects, in this paper STFT, the one allowing uniform division of the *TF* space, was used and an approach to quantify information of the spectrograms based on multiple features extraction was presented. To process the analysis of information content of *TF* representation, the BN signals obtained during tensile loading of the steel samples were used. The loading process introduces in the material changes of the mechanical structure, which affect the magnetic properties and further reflects in the dynamics of Barkhausen effect [4,7,10,24,44]. Thus, application of different stress level allows to observe the changes in the BN signal dynamics. First, in order to adjust transformation properties, the measurements were conducted under various magnetization conditions along with several setups of the transform. The results were shown and discussed. Then *TF* feature extraction procedure was presented and conducted for successive loading force levels. Finally, in order to validate the information content, the obtained distributions of *TF* features were compared with the ones obtained during classical analysis method (carried out separately in *T* and *F* domain). The results are presented and discussed.

## 2. Experimental Setup and Measurements

**Figure 2:**
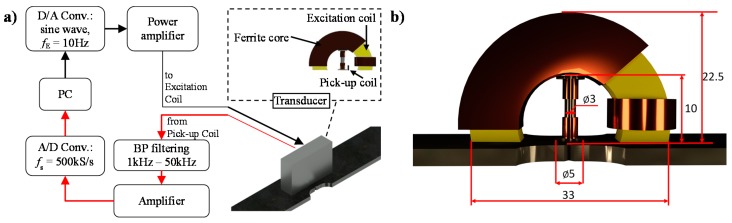
Measuring system setup: (**a**) the block diagram, (**b**) view with dimension of transducer.

All data used in the experiments were acquired during stress loading tests of the steel samples. The measurements were carried out using a computerized system. The block diagram of the system is presented in Figure 2. The observation of MBN was processed using a transducer containing two subunits: those for magnetization and acquisition. The sample was magnetized using a field generated by a coil wound on C-shaped ferrite core. The BN signal was gathered by a pick-up coil section wound on a rod ferrite, placed between the pole pieces of the magnetizing section’s ferrite core. Before the acquisition process, the BN signals were filtered (1 kHz–50 kHz) and amplified. The observation of the Barkhausen effect is carried out usually for frequency of a magnetization field ranging from tenths to several tens of Hz. However, at lower bands the reduced intensity of the effect is obtained. At the same time, the concentration of the domain reorientation phenomenon at a shorter time along with increase of eddy current damping has an impact at higher bands. Thus, the highest sensitivity of features to materials changes can be obtained for middle bands of the frequency range [25]. Therefore, during the tests, the excitation coil of the BN transducer was driven with a sinusoidal waveform with a frequency *f*_E_ of 10, 20 and 30 Hz. Three frequencies were used in order to observe the influence of *f*_E_ on parameters distributions under different sampling resolution conditions. The pick-up signals were acquired during five magnetization periods using NI USB-6251 DAQ device with a sampling frequency *f*_s_ of 500 kHz. All measurements were conducted during the stress loading process.

**Figure 3:**
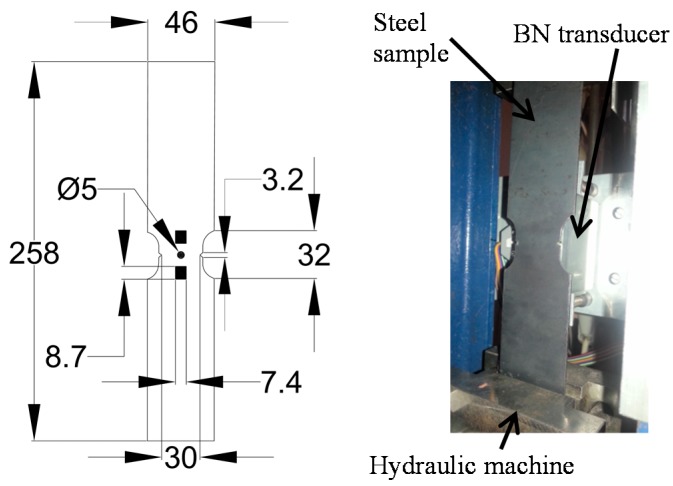
View of the sample shape with dimensions in [mm]: 2D model and photo. The samples’ thickness is 2 mm.

**Figure 4:**
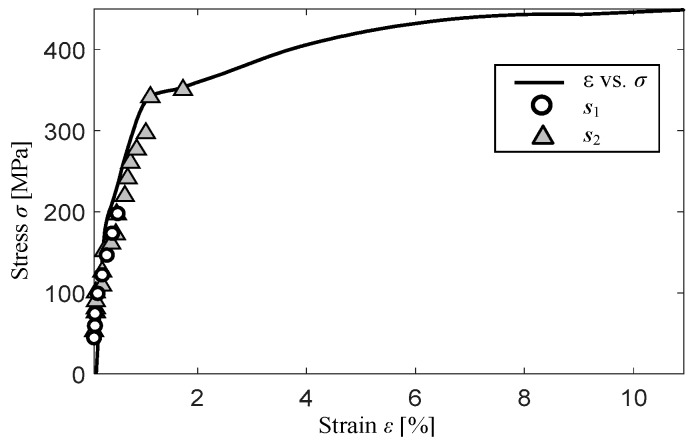
Stress-Strain curve obtained during tension of the used steel with visualization of successive measuring points for two presented samples *s*_1_ and s_2_.

The experiments were carried out for samples made of St3S (according to standard PN-88 H-84020) low-carbon steel, commonly used for various constructions. During the measurements, the transducer was picking-up the signal at a single position over the sample. Therefore, in the middle part, where the transducer was fixed, the sample was narrowed to concentrate the material changes in this region. The shape with dimensions of the sample along with the visualization of transducer’s position was presented in Figure 3. To observe different damage stages in the material, the samples were subjected to stress σ (tension) ranging from 0 to 350 MPa (0 MPa refers to as-cast state). The yield point of the used steel grade is in the range of 185–235 MPa, while the limit state in 340–520 MPa. The stress-strain curve of the steel, with depicted levels at which the measurements were carried out for two testing samples *s*_1_ and *s*_2_, is shown in Figure 4. Both samples were loaded using different tensile force limits. The sample *s*_1_ was tensile loaded within the elastic range, while in case of the sample *s*_2_ the tests were continued up to the stress level over the steel’s yield point. Consequently, there were more observations data points for sample *s*_2_ than for *s*_1_ one.

## 3. Time-Frequency Domain Analysis of MBN

The STFT-based *TF* representation is mostly used for spectral analysis and processing of non-stationary signals under various applications for e.g., audio signals [45], medical EMG and EEG signals [46] or for speech recognition [47]. The development of accurate decomposition of signals, complex in time or space, allows showing the variability of amplitudes, frequencies and phases of component signals. For that reason, time-dependent multispectral analysis is being applied by decomposing an input signal using a sliding window in *T* domain. In the following section, the properties of STFT in terms of BN signal analysis are discussed. Several aspects should be considered before performing the computations. The transformation parameters defining the frequency and time resolutions as well as magnetizing field frequency were considered in the process. Then the computations were performed and the results presented.

### 3.1. STFT-Based TF Representation

The short-time Fourier transform is characterized by the high redundancy of information contained in the *TF* representation. The distribution is composed of the Fourier transform results obtained for subsequent time periods of the signal [48]. Each time a given signal interval and a window function is considered. The STFT is defined by the general expression [48]:

(1) $$X(t,f)=\int_{-\infty }^{\infty }x\left(\tau \right)w(\tau -t)e^{-j2\pi f\tau }d\tau$$

where *x* expresses an analysed signal, *X* is a complex result of STFT transformation and *w* is a window function. In case of digital signal, a discrete form can be applied [48]. Next, based on the given definition, a spectral density function *SPEC*(*t*, *f*) called spectrogram can be defined as:

(2) $$SPEC(t,f)={\left|X(t,f)\right|}^{2}$$

It can be noticed that the transformation results, beside the analysed signal itself, depend on a type of window function *w* and its length, by which the signal is multiplied prior obtaining Fast Fourier Transform (FFT). Therefore, in order to properly process the analysis, it became important to choose the appropriate type of window *w* function and to adjust its parameters as well. There are many functions representing polynomial, cosine-sum and adjustable type of characteristics [48]. When analysing the properties of a given functions, two parameters can be considered of the frequency representations: the main-lobe width and side-lobe area. The rectangular window is the simplest nonparametric window type. In discrete form it assumes the value equal to 1 over whole its length 0 ≤ *n* ≤ *N* − 1 and 0 in other case (*n* is a given sample and *N* is the length of the window). It is mostly used in cases when the energy of the analysed signal is distributed unevenly in time. Its characteristic is described as the one with low dynamic range. Therefore, to improve the dynamics range, other windows are defined such as polynomial, cosine-sum or adjustable ones. An example of polynomial function is a triangular window, while the Hamming window represents the cosine-sum type of function. Those are non-parametric types of windows. However, in case of a system that requires changes of measuring conditions, the adjustable window type can be beneficial. In this case, a Kaiser window can be used. The function minimizes the width of the main lobe and the percentage of energy of the side lobes at the total spectrum energy [48]. The Kaiser window has the following form:

(3) $$w(n)=\frac{I_{0}\left(\beta \sqrt{{\left(\frac{n-\frac{N}{2}}{\frac{N}{2}}\right)}^{2}}\right)}{I_{0}(\beta )}$$

where the *I*_0_ is the Bessel function of zero order. The value of *β* parameter was set to 0.5 in the presented case. The application of various window functions affects the STFT results. The influence of the window type and its length on *TF* distribution of BN signal is presented in next section.

### 3.2. TF Transformation Configuration for BN Signals Analysis

**Figure 5:**
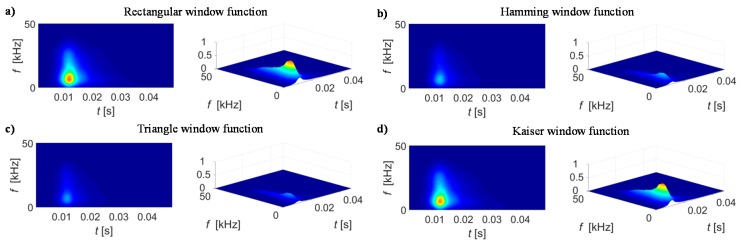
Spectrogram results obtained using window of 512 samples defined by function: (**a**) rectangular, (**b**) Hamming, (**c**) triangular, (**d**) Kaiser. The excitation and sampling frequency were equal respectively 10 Hz and 500 kHz.

**Figure 6:**
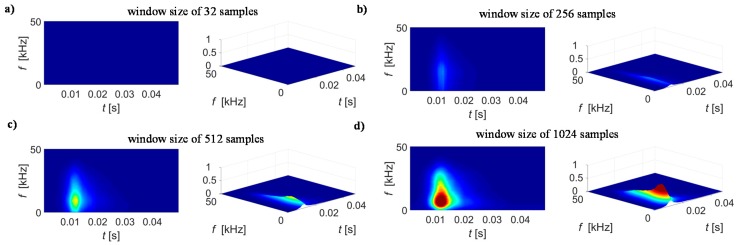
Spectrograms obtained for window size of: (**a**) 32 samples, (**b**) 256 samples, (**c**) 512 samples, (**d**) 1024 samples.

**Figure 7:**
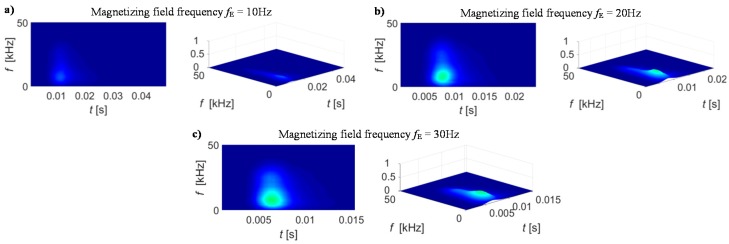
View of spectrograms for different magnetizing field frequency *f*_E_: (**a**) 10 Hz (**b**) 20 Hz (**c**) 30 Hz.

In addition to the window type selection, the measuring condition defined by the sampling frequency and its ratio to the magnetizing field frequency should also be taken into account as this affects also the desired window length and the sensitivity of the STFT results. Therefore, in order to adjust the STFT transformation conditions, the preliminary tests were carried out. Those allowed calculation of STFT representations of BN signal *S*_BN_(*t*, *f*) and further theirs spectrograms |*S*_BN_(*t*, *f*)|^2^. Consequently, the qualitative assessment was performed. As the signals were acquired each time for five magnetization periods, ten spectrograms were obtained for each case. This allowed application of the smoothing procedure which resulted in calculation of the average |*S*_BN_(*t*, *f*)|^2^ distribution denoted as *BN*_TF_S_. The results of *BN*_TF_S_ obtained for selected MBN signal under various transformation parameters are presented in Figure 5, Figure 6 and Figure 7.

First, the influence of a window type on the spectrogram distribution was considered (Figure 5). The distributions (achieved for *f*_E_ of 10 Hz, *f*_s_ of 500 kHz and *N* of 512 samples) obtained in case of rectangular and Kaiser windows presents higher rate of distinguishability between the different levels of energy distribution. The Kaiser window function allows conducting the optimization process and thus is more flexible for various conditions. The window can be adjusted by changing the *β* (Equation (3)). By setting *β* to 0 the rectangular window can be obtained, while with increasing the *β* value the curve distribution narrows down in time domain and simultaneously the lower level of side lobes in frequency domain are obtained. Therefore, taking into consideration all those aspects, the Kaiser type of window was used in further analysis.

In the second stage, the analysis of the spectrogram *BN*_TF_S_ resolution was carried out. It must be noticed that there is a strict relationship between the representations resolutions in time and frequency domains. If the time period Δ*T* is increasing, the frequency resolution Δ*F* is simultaneously decreasing [49,50]. The best resolution is maintained when Δ*F* = 1/Δ*T*. There are works on the methods for optimization of time resolution Δ*T* [49,50]. However, these mostly concern the linear frequency modulated signals. In other cases, usually the procedures follow selection of window length from a pool of window sets [49]. For that reason, several window sizes were used ranging from 32 up to 1024 samples, what corresponds respectively to time periods from 64 µs to over 2 ms. This results in expecting the Δ*F* to be in the range of 15625 Hz and 488 Hz for the shortest and the longest Δ*T* respectively. Taking into consideration that the BN signal is with noise-like wideband frequency characteristic it is required to apply high resolution Δ*F*, in this case equal to 488 Hz. This would result in insufficient time resolution. Therefore, to overcome this aspect, in further calculations, the window overlapping technique was used with the overlapping step of 0.75. Consequently, it was possible to obtain the effective Δ*T* equal to 0.5 ms. Considering the results presented in Figure 6, one can clearly notice that the greatest visibility of energy distribution was obtained for the largest size of the window. In this case, the time period allows for observing a significant number of BN signal peaks (MBN activity). In the same time, taking into consideration the bandwidth of the measured BN signals (between 1 kHz and 50 kHz) the frequency resolution is still enough to observe the details of changes in frequency characteristic and thus further increasing of Δ*F* would not affect much the results. Therefore, in the later experiments the window size of 1024 was applied.

In the final test, in order to verify qualitative influence of the measuring conditions on the resulting *BN*_TF_S_ spectrograms, different excitation signal frequencies of 10 Hz, 20 Hz and 30 Hz were applied during the signal acquisition. It can be noticed that the increase of the excitation frequency also caused the change in energy distribution (Figure 7). The higher rate of fluctuation of magnetizing field direction forces the domains to rotate at shorter periods. Therefore the majority of energy in the *BN*_TF_S_ spectrogram is concentrated in the vicinity of the beginning of the magnetic field period. As the resolution in time decrease with the increase of the excitation frequency, a higher number of BN peaks can be observed for each window. Thus, in order to gain the highest possible discrimination level between the different states of the examined sample, in the following stage, the highest sampling frequency and the lowest excitation frequency was further used.

### 3.3. Procedure for Features Extraction Based on TF Representation

**Figure 8:**
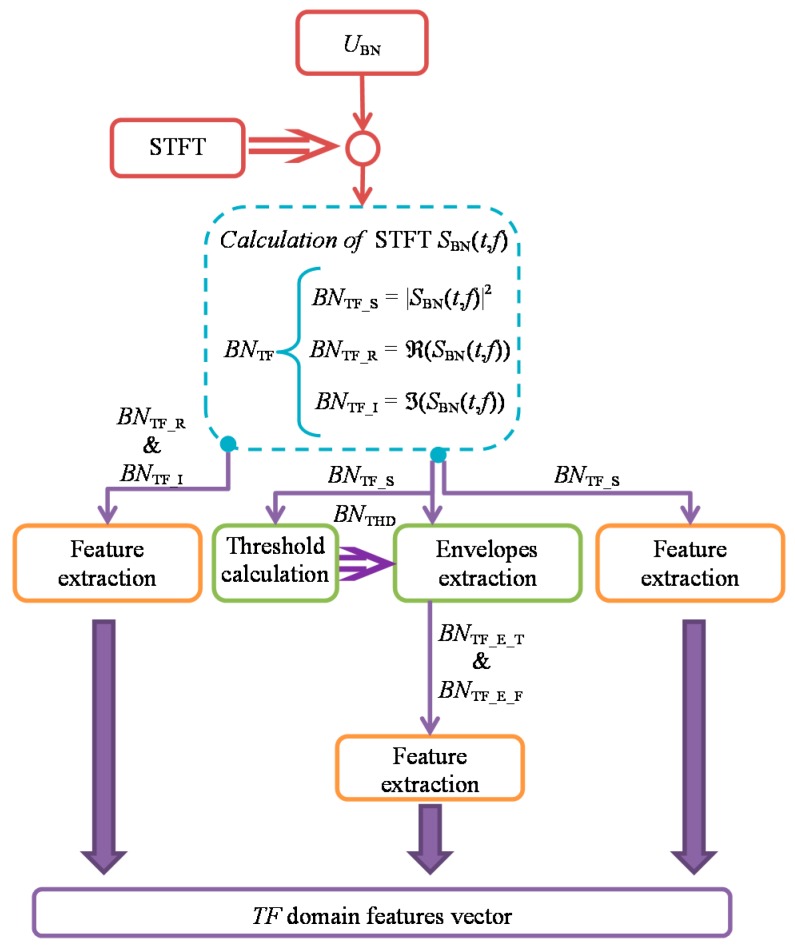
Diagram of the feature extraction procured applied to *TF* representation of BN signals.

Use of *TF* distributions in their entirety for non-destructive assessment of changes of material states would be computational challenging process requiring to use e.g., a highly complicated multiple-layers artificial neural network [51,52,53,54,55]. Thus, in order to describe variations in the achieved characteristics, and on that basis processing in the future e.g., the classification, the feature extraction was implemented. Considering the need for characterization of *TF* representation of BN signal, a set of features has been calculated. The general diagram of the proposed feature extraction procedure was presented in Figure 8. The exploration of the *TF* representation *S*_BN_(*t*, *f*) was carried out for three forms. First the spectrogram was analysed. Then, the method allowing delineation of the greatest activity part of the spectrograms by two envelopes over *T*- and *F*-axis was proposed and implemented. Finally, the real and imaginary parts of the spectral density *S*_BN_(*t*, *f*) were also analysed.

To conduct the first form of the computation procedure, the 2D spectrogram *BN*_TF_S_ was considered. The essence of BN representation analysis is the determination of several parameters enabling the observation of *TF* distribution changes under any aspect. The key role in later interpretation may depend on the detection of variance in the dynamics of energy distribution, concentration, centroid shift in time and frequency, and the degree of disorder or scope of changes. These aspects are affected by changes in magnetic properties forced by material changes that occur as a result of several different factors, such as change in the stress state and depth of hardening. There are many features used in audio signal recognition and classification or biomedical applications [56,57,58,59,60]. Those can be useful for BN signal analysis; however, they have not found much interest in previous research. Generally, the spectrogram features are defined by extending *T* and *F* domain features definition over the common 2D *TF* spatial representation [56,57,58,59,60]. This allows obtaining additional information which would not be available under single domain representation regime. They mostly refer to some well-known statistical properties applied to the *TF* representation such various forms of mean values (i.e., arithmetic, geometric, harmonic or generalized) centroid, variance or standard deviation, skewness or kurtosis. Other aspects being analysed are the shape of the *TF* spectrogram, its energy distribution or entropy. The shape related features allow distinguishing between the signals with slow and fast varying spectral content. The others express the uniformity of the energy distribution and randomness in the distribution of signals energy. For shape assessment of *TF* distribution, the *TF* flux, spread or slope are frequently used. The flux rates the amount of the changes of signal energy and the spread describes the concentration of power around spectral centroid while the slope measures the slope of the TF representation shape [56,57,58]. The energy concentration level can be evaluated on the basis of spectral flatness or concentration measures [56,60]. The first one expresses the ratio between the geometric and the arithmetic mean of *TF* what results in high value of coefficient for uniform distributions. The example for the second one is the Minkowski distance, which reaches higher values when the energy is spread out over whole *TF* plane [56]. The last crucial aspect is the assessment of the degree of order/disorder of the *TF* representation. For that purpose the entropy is calculated.

Taking into consideration all presented circumstances, in order to extract the information content which further can be useful for evaluation of changes of BN signal’s *TF* representation under all mentioned aspects, several spectrogram features were chosen and used. To clarify the calculations process, the definitions of the features are presented in Table 1. Finally, as a result of conducted calculations, a set of variables were obtained referring to spectrogram’s characteristic values such as maximum (*BN*_TF_MAX_) and its location on time and frequency axis or mean, standard deviation and variance as well. To assess the changes of the energy distribution concentration the spectral flatness *BN*_TF_SF_, the spectral entropy *BN*_TF_SE_, the concentration measure *BN*_TF_CM_ and the coefficient of variation *BN*_TF_CoV_ were calculated [56,57].

**Figure 9:**
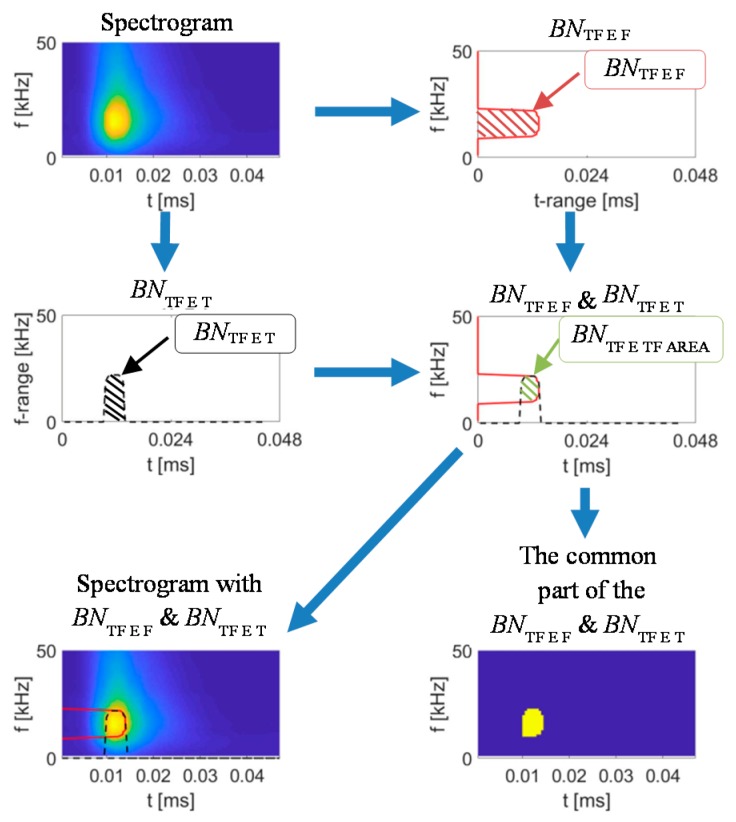
Visualization of the envelopes extraction procedure.

The proposed in this paper, second form of the computational procedure refers to extraction of instantaneous features being a 1D functional representation of 2D spectrogram *BN*_TF_S_. It is based on the calculation of two 1D curves over *T* and *F* axis, denoted as envelopes *BN*_TF_E_T_ and *BN*_TF_E_F_ (Figure 9). The envelopes over the *T* and *F* axis ware obtained by delineation of area along each axis, where the value of the spectrogram is not lower than the requested threshold *BN*_THD_, for a given block range (computational cell) of *F* or *T* values. For example, in case of extraction of *T* axis envelope, at each time step (computation cell) *t_i_* the values over the whole *F* axis were compared with the threshold *BN*_THD_:

(4) $$BN_{\mathrm{TF}\_E\_T}\left(t_{i}\right)=\left\{\max\left(f_{j}\right)|BN_{\mathrm{TF}\_S}\left(t_{i,}f_{j}\right)\geq BN_{\mathrm{THD}}\right\}$$

where: *i* = 1…number of time steps, *j* = 1…number of frequency bins (FFT points).

The highest value of *j* for which the above condition is true defines the frequency bin value for a given time step *i*. According to the described procedure, the envelope over the *F* axis was also defined. Then, the fitted curve was further analysed to obtain the set of standard statistical values and the one describing the curve’s shape (e.g., skewness *BN*_TF_E_SKEW_ or kurtosis *BN*_TF_E_KURT_) as well. Additionally, the common area of both envelopes delineates the area of the greatest activity depicted as a *BN*_TF_E_TF_AREA_ and was considered in the feature extraction procedure. The envelopes *BN*_TF_E_ features were defined analogically to the ones presented in Table 1, by considering only one dimension during calculations. The type of the envelope (along *T* or *F* axis) from which a given feature was calculated was denoted then by letter T or F by its name. The whole computation process of the used during the final experiment threshold value was described in the next section of the paper.

Not only the magnitude may carry important characteristics while the phase change of the STFT representation can provide one with additional information as well. Therefore finally, in the process of extraction of complementary knowledge, the calculations were also carried out for both real *BN*_TF_R_ and imaginary *BN*_TF_I_ parts of the initially obtained 2D transformation *S*_BN_(*t*, *f*) of MBN signal. The set of features were obtained based on same procedures used prior for the spectrograms *BN*_TF_S_ distributions.

At the end, the extended *TF* feature vector was obtained allowing evaluation of various aspects of *TF* properties changes. Consequently, this can enhance the overall perception for monitoring of mechanical changes of the examined material. The evaluation of the quality of the extracted features was then performed for a set of signals acquired during stress loading of steel sample.

## 4. Stress Evaluation Experimental Results and Discussion

To evaluate the effectiveness and quality of the information extracted from the *TF* representation, the verification experiment was carried out. The main idea was to monitor the changes of the magnetic conditions under the changes of mechanical properties of the examined material. For that purpose, the stress loading experiment was carried out and a set of BN signals was acquired. Next, the evaluation of the *TF* representation quality was processed under two stages: qualitative and quantitative assessment. Finally, in order to verify the information content of the *TF* representation, the obtained *TF* features distributions were complied with the commonly used BN signals features extracted from single *T* or *F* domain.

### 4.1. Qualitative Evaluation of Spectrogram Changes under Progressive Stress-Strain Condition

**Figure 10:**
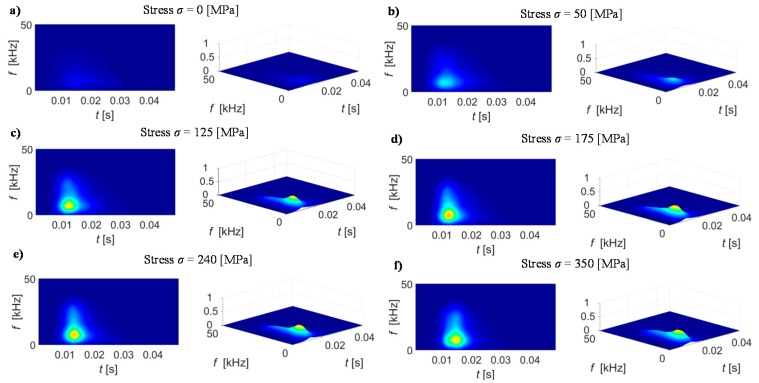
Comparison of the spectrogram in terms of different applied stress level (in [MPa]): (**a**) 0, (**b**) 50, (**c**) 125, (**d**) 175, (**e**) 240, (**f**) 350.

First, the obtained during preliminary experiments configuration of STFT transformation conditions were used to calculate the *TF* representation *S*_BN_(*t*, *f*) of BN signal acquired for steel samples at various stages of the stress loading process. The selected results of spectrograms *BN*_TF_S_ obtained for sample *s*_2_ for applied different stress level are presented in Figure 10. It can be noted that in case of as-cast state (no loading force) the spectral density in *BN*_TF_S_ presents rather uniform distribution of over whole *TF* plane and reaches relatively low value (Figure 10a). Then, even low stress, introduced in the sample during tensile loading, results in the rise of the energy level emanated within the low frequency range (up to 10 kHz) and close to the beginning of the MBN burst (peaks activity) period (Figure 10b). Further increase of the stress level, to the one close to yield point, resulted in rising of the activity of MBN also in the higher frequency range (Figure 10c,d). For the stress level close to the limit state, one can observe the shift of the high activity region in time towards the time span referring to occurrence of the maximum of the magnetization field (Figure 10e). Further increase of the tensile force causes further shift of the high-energy area along with a slight decrease of the highest spectral energy level, what can be noticed in case of the results obtained at plastic deformation level (Figure 10f). The observed effect can be explained by referring to the magnetic domain theory [1,34,35]. Because the direction of the magnetization is consistent with the direction of the applied stress in the examined samples, the increase in the tensile load induces a reorientation of the magnetic domains. First, the orientation towards the axis of easy magnetization closest to the axis of applied stress takes place, followed by orientation towards the axis of the applied stress itself. As a result, domains magnetized in the direction parallel to the stress will increase their area at the expense of domains oriented in other directions. Taking into consideration that the generation of Barkhausen noise is closely related to the reversal of magnetic domains with a magnetization direction parallel to the excitation field, the increase in these domains as a result of tensile stress increases the MBN activity [34,35]. As the lower magnetic field is required to force the reorientation under tensile stress, MBN signal shifts towards the lower magnetizing field value [39]. This state dominates within the elastic tensile stress range (Figure 10a–d). Therefore, the initial growth of the highest energy area on spectrograms is noticeable. As part of these changes, attention should be paid to two aspects. The first is the increase in the maximum value, while the second is the increase of the frequency band and the extension of time range. Additionally, it can also be observed that the *BN*_TF_S_ distribution is not symmetric with a rapid growth of energy at the beginning of the reorientation period (with maximum value occurring close to it) and its smooth fall in the further part of the time. In the time domain, these changes are associated with a simultaneous increase in the MBN signal envelope’s peak height along with decrease of its width, shift of its peak position, as well as with increase of its skewness (right-skewed distribution). Those observations find confirmation in other works [36,37,38]. Growth of the amplitude and decrease of the width affect both the maximum value and the frequency band of the spectrogram distribution. The higher value of MBN signal distributed in shorter time results in higher energy and wider bandwidth in *BN*_TF_S_. Similar conclusions can be drawn for other discussed aspects. In case of higher tensile stress regime the plastic deformations are introduced creating a increase in dislocation density resulting in formation of pinning sites blocking the free movement of the magnetic domains walls, which significantly weakens the aforementioned reorientation process [35,36,37]. Therefore, the MBN activity is reduced finding its reflection in MBN signal changes. The peak height as well as the MBN signal skewness is slightly decreasing [36]. As higher energy state is required for domains to overcome the obstacles, the shift of the peak position to higher magnetizing field value is also reported [36,39]. Consequently, the spectrogram changes can be also observed (Figure 10e,f) e.g., in the form of decrease of maximum value of *BN*_TF_S_ or its shift in time.

The achieved spectrogram distributions confirmed the usability of the information content of the *TF* representation in the process of stress evaluation. It is possible to clearly notice the influence of the material properties changes based on the achieved *TF* distributions.

### 4.2. Quantitative Evaluation of TF Domain Information under Progressive Stress-Strain Conditions

**Figure 11:**
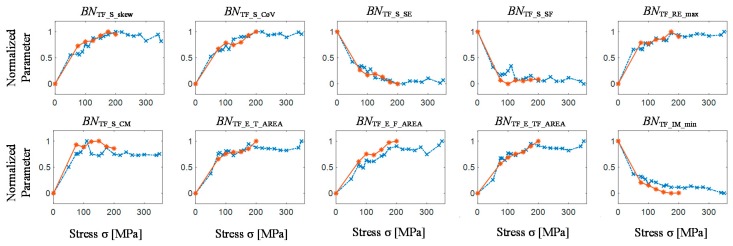
Distribution of selected features extracted from of *TF* representation of BN signal for successive stress-strain stages; sample *s*_1_ results—red line; sample *s*_2_ results—blue line.

The information preserved in the *TF* representation of MBN is not apparent and, as it was discussed in the previous section, is reflected in various forms of distribution changes. Use of only some basic characteristic values do not allow full evaluation of the observed variations in the dynamics of the process. Therefore, in order to enable evaluation of the quality of information provided by *TF* distributions in a broad aspect, a set of features representing all three forms of the proposed calculation procedure (expressing statistical properties, shape, energy distribution and disorder degree along with the properties of extracted features) were selected for the final stage. To determine the threshold value necessary to extract the envelope over the *T* and *F* axes, the results of the analysis carried out for the first form of representations (i.e., spectrograms of measured BN signals) were used. Considering the changes in the spectrogram distributions that were observed during the first part of the final experiment, the threshold value was determined based on the results obtained for the unloaded as-cast sample (the reference sample—with the lowest level of obtained values). First, the spectrograms for BN signals acquired during several periods of the magnetizing field were calculated and the maximum values *BN*_TF_S_MAX_ were determined for each burst of the analysed BN signal. Then, the interquartile range for the series of obtained values was determined and the mean of only those values, which were within that range, was calculated. The final threshold value *BN*_THD_ corresponds to that value reduced by the square root of two (which corresponds to 3 dB on a logarithmic scale). Results of the selected *TF* features distributions as the function of obtained stress level are presented in Figure 11 (see also Table 1). All results for *s*_1_ and *s*_2_ sample were normalized to 0–1 range in order to compare the behaviours of distributions. As one can see, the distributions achieved for both samples present similar behaviour what confirms the repeatability of the applied procedures. For all presented characteristics, the greatest variation of value can be observed between the as-cast state and the first stage of loading process. This is fully in agreement with the results of the qualitative observation of spectrogram changes, conducted during the first stage of the assessment (Figure 10). Generally, the features follow monotonic changes over the observed stress range. Only in case of concentration measure *BN*_TF_S_CM_ the value initially increases and then mostly decreases. It can be noticed that the entropy of the power distribution *BN*_TF_S_SE_ decreases with the increase of the stress state, which can be understand as leading of the *TF* representation to higher degree of order and to accommodate the existing energy states. Observed dependencies are associated with the increase of MBN activity within the elastic stress regime, which was discussed in the previous section. At the same time, there is an increase in the difference in values between the occurring states. The value of the standard deviation increases much faster than the average value, which is reflected in the increase in the coefficient of variance *BN*_TF_S_CoV_. The observed phenomenon is also visible in the decrease of the course of spectral flatness *BN*_TF_S_SF_. The spectral flatness represents the geometric mean of *TF* representation to its arithmetic mean, what results in achieving higher values of *BN*_TF_SF_ for randomly distributed signal. Therefore, in reference to the presented spectrogram changes, as the distribution with progress of the stress-strain stage is getting more consistent, the *BN*_TF_SF_ is decreasing. However, it should be noted that after a decrease in the *TF* distribution concentration for low stresses below 120 MPa (increase in the *BN*_TF_S_CM_ value), its smooth increase is visible for higher load states. The confirmation of this fact is the initial increase and then the slight decrease of the area under the envelopes *BN*_TF_E_T_AREA_ and *BN*_TF_E_TF_AREA_. Although monotonic changes in the observed characteristics can be seen, one can indicate precisely the area in which the change of local trend takes place. This refers to the stage of the process related to the yield point area of the used steel (close to 200 MPa). This depicts the border between the elastic and plastic regime and the change in the MBN activity which was discussed in the previous section. The above description can be noticed on the courses of maximum of real *BN*_TF_RE_max_ and minimum of imaginary part *BN*_TF_IM_min_ of the spectral function. Similarly, it can be observed on all three envelopes areas *BN*_TF_E_T_AREA_, *BN*_TF_E_F_AREA_ and *BN*_TF_E_TF_AREA_, spectral entropy *BN*_TF_SE_, skewness *BN*_TF_S_skew_ and coefficient of variance *BN*_TF_S_CoV_ distributions as well. However, the sensitivity for this effect varies within the features set. This can be beneficial under terms of future classification procedures.

Although the influence of applied strains on the Barkhausen effect is widely studied, its characteristics depend on many factors. In particular, the impact of plastic deformations after exceeding the yield point on MBN is considerably complicated and still not very well understood [5,6,7,11,29]. The interpretation of the observed characteristic in reference to the magnetic structure behaviour is not straight and requires additional material examination experiments. However, the obtained *TF* features distributions allow clearly monitoring the progress of the stress-strain process.

### 4.3. Comparison of TF, T and F Features Distribution

To validate the information content of *TF* representation for the need for non-destructive estimation of loading state, the *TF* features distributions were compared with the well-known ones, commonly used and allowing characterization of BN signal in single domain [12,22,24,36,61]. Therefore, signals acquired during the experiments were processed first in the *T*, and then in the *F* domain. In case of the *T* representation, statistical and characteristic quantities were computed directly from the obtained BN signal *U*_BN_. As a result, features such as peaks number (*BN*_N_), peak-to-peak value (*BN*_Vpp_), root-mean-square (*BN*_RMS_) or energy (*BN*_EN_) were obtained. Additionally, the BN signal’s envelope was also considered allowing calculations of a set containing features such as peak value, position (*BN*_l_MAX_) or width, as well as interquartile range (*BN*_IQR_), skewness or kurtosis (*BN*_KURT_). Furthermore, features expressing ratio between various characteristic quantities of *T* representation such as crest and impulse factor were also calculated. The crest, also called peak-to-rms factor, is defined as [61]:

(5) $$BN_{\mathrm{CREST}}=\frac{0.5\cdot BN_{\mathrm{Vpp}}}{BN_{\mathrm{RMS}}},$$

while impulse, frequently used to characterize electrical signals also called form factor, as:

(6) $$BN_{\mathrm{IMPULSE}}=\frac{BN_{\mathrm{RMS}}}{\frac{1}{N}\sum_{i=1}^{N}\left|BN_{i}\right|}.$$

For *F* representation analysis, Fast Fourier Transform was used. The frequency spectrum characteristics were analysed and the features set corresponding to the set obtained for BN envelope was achieved (e.g., maximum of spectral power density *BN*_f_PMAX_, its position of *F* axis *BN*_f_fMAX_, or standard deviation of spectral power density distribution *BN*_f_STD_, etc.). The details of the applied features extraction procedure can be found in [12,22,24]. The usability of all those features for stress or hardening level evaluation was also confirmed by other works [14,34,36,61].

**Figure 12:**
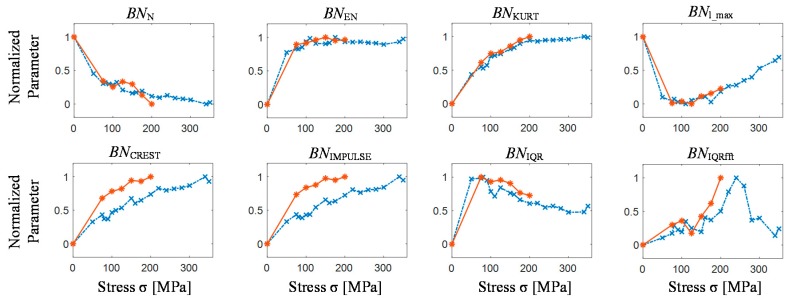
Features distribution extracted from *T* and *F* domain representation of BN signal for successive stress-strain stages; sample *s*_1_ results—red line; sample *s*_2_ results—blue line.

Selected results of the features obtained from *T* and *F* representations are presented in Figure 12. Also in this case, one can observe a significant change in the value of individual features between the as-cast state and the first level of the loading process. The number of peaks successively decreases with the growth of stress level (*BN*_N_), while simultaneously the energy of the pulses *BN*_EN_ is increasing up to the stress level close to 200 MPa end then starts to slightly decrease. The observed relationship (lower number of events resulting in similar cumulative energy) is in good agreement with the observations conducted in Section 4.1. The initial increase in MBN activity results in superposition of single MBN pulses to form one of higher amplitude. Consequently, the number of pulses decreases while the energy increases. This confirms the decrease of *BN*_TF_S_SE_ and also of *BN*_TF_S_CM_ presented at *TF* features distributions results (Figure 11.). Furthermore, the shift of peak position *BN*_l_MAX_ is also clearly visible. First, as the lower external energy is needed to process the reorientation of magnetic domains for elastic stress regime, peak position moves to the times referring to beginning of the domain reorientation process. Then within the range of around 100‒200 MPa it stabilizes and finally, when the pinning sites effect intensifies, it shifts toward the centre of magnetization period. The interquartile range of BN signal’s distribution in time *BN*_IQR_ after initial increase beyond the 100 MPa starts to decrease, which indicates that the signal is getting more concentrated. Slightly different characteristic can be observed for interquartile range of frequency spectrum characteristic. The *BN*_IQRfft_ generally increases to around 230 MPa; however the change of the dynamics can be noticed over 180 MPa level. Above the limit of 230 MPa, the feature value starts to monotonically decrease. This corresponds to the extension of the frequency bandwidth visible in the spectrograms achieved for elastic stress regime. The crest and impulse factors depicting the relationship between the peak-to-rms and expressing shape of the waveform is systematic increasing. This can be referred to the constant increase of difference between the highest MBN pulses and the medium ones (the difference between the *s*_1_ and *s*_2_ sample occurs as a consequence of normalization procedure). This finds confirmation in kurtosis as its dynamic of increase is lower above 200 MPa aiming saturation. This means that majority of impulses of the MBN signal are more concentrated in time. Nevertheless, all obtained characteristics are in agreement with the results presented in other works [34,35,36,37,38,39,61] and confirm the observation carried out for spectrograms and *TF* features distributions. For example, the change of the shape and variation in peaks energy distribution was revealed in *BN*_TF_S_Skew_, *BN*_TF_S_SF_ and *BN*_TF_S_CoV_ features plots and was also noticed in *BN*_KURT_, *BN*_CREST_ or *BN*_IMPULSE_ features courses as well.

## 5. Conclusions

In this paper, the time-frequency representation was used to analyse the information content of Barkhausen noise BN signal in terms of use for non-destructive evaluation of material state and properties. To process the analysis, the STFT transformation was applied. First, several aspects of the transformation setup were analysed and the influence of the various configurations on *TF* distributions properties of BN signal was shown and discussed. Consequently, the transformation parameters along with the measuring conditions (i.e., magnetizing field frequencies) were adjusted and used during further work. Then, in order to quantify the information content provided by *TF* representation, the feature extraction procedure was introduced. Although the *TF* representation of the BN noise contains a lot of information that can be used in the process of non-destructive evaluation of materials, little work has been done to quantify it. Therefore, the purpose of this paper was to carry out a wide analysis allowing obtaining a large representation of extracted features expressing the observed changes in a broad context. The process includes different forms of the obtained STFT representations.

To evaluate the information content of the *TF* representation, finally, the analysis was carried out for a set of BN signals acquired during non-destructive evaluation of steel samples. To introduce changes of material properties, the samples were tensile loaded during the measurements. Then the resulting spectrograms were shown and qualitative evaluation of their properties was processed. Next, in order to quantify the observed properties, the proposed feature extraction procedures were applied. Consequently, it was shown that analysis of the *TF* domain spectrograms of BN signals provide one with valuable information which allows to discriminate the various state of the material subjected to stress. The analysis of many features of the obtained *TF* distributions enables effective observation. The achieved characteristics between the parameter value and stress can, consequently, improve the accuracy of proper identification of the state of the structure under examination. To validate the information content preserved by the *TF* plane, the *TF* features distributions were compared with the one commonly used in various MBN applications derived from single *T* and *F* domains. The achieved results allowed to draw similar conclusions and confirmed the usability of the *TF* representation in the material evaluation procedure. The similarities in information content of features extracted from all three domains were noticed. As the presented *T* and *F* features have already proved their effectiveness in MBN signal analysis in many works, the achieved results confirms the possibility of using the *TF* representation for the need for efficient evaluation of material properties. In addition, the *TF* plain can become beneficial. The representation can be useful especially when assessing the material properties which vary i.e., within its depth. The use of the *TF* representation enables the analysis of signals over 2D plane, thus it is also possible to observe changes in both domains simultaneously. Therefore, information encoded in time and frequency is more accessible, furthermore allowing analysis of the temporal change of dynamics. The dynamics of changes is closely related to the course of the magnetic domain’s reorientation process, thus it creates the possibility to obtain new or supplement information expressed in single domains of time and frequency. In addition, the integrated *TF* domain allows combining methods of analysis and unification of calculation procedures. Consequently, it is believed this may lead to a more robust and stable evaluation of the condition and properties of the material.

In the future, it is planned to conduct various experiments for non-destructive assessment of depth-varying properties such as case hardening and to study this behaviour. Furthermore, additional methods for the analysis of time-frequency representations are going to be implemented. It is also planned to use the obtained features set to build a multi-parameter classification model.

## Figures and Tables

**Table 1:** Definition of selected features calculated based on spectrogram *BN*_TF_S_ of *TF* representation *S*_BN_(*t*, *f*) ^1^.

Feature	Formula
Maximum	$BN_{\mathrm{TF}\_S\_\mathrm{MAX}}=\max\left\{BN_{\mathrm{TF}\_S}\right\}$
Mean	$BN_{\mathrm{TF}\_S\_\mathrm{MEAN}}=\frac{1}{N\cdot M}\sum_{i=1}^{N}\sum_{j=1}^{M}BN_{{\mathrm{TF}\_S}_{i,j}}$
Standard deviation	$BN_{\mathrm{TF}\_S\_\mathrm{SD}}=\sqrt{\frac{1}{N\cdot M}{\sum_{i=1}^{N}\sum_{j=1}^{M}\left|BN_{{\mathrm{TF}\_S}_{i,j}}-BN_{\mathrm{TF}\_S\_\mathrm{MEAN}}\right|}^{2}}$
Variance	$BN_{\mathrm{TF}\_S\_\mathrm{VAR}}={\left(BN_{\mathrm{TF}\_S\_\mathrm{SD}}\right)}^{2}$
Skewness	$BN_{\mathrm{TF}\_S\_\mathrm{SKEW}}=\frac{\sum_{i=1}^{N}\sum_{j=1}^{M}{\left(BN_{{\mathrm{TF}\_S}_{i,j}}-BN_{\mathrm{TF}\_S\_\mathrm{MEAN}}\right)}^{3}}{N\cdot M\cdot BN_{\mathrm{TF}\_S\_\mathrm{SD}}{}^{3}}$
Kurtosis	$BN_{\mathrm{TF}\_S\_\mathrm{KURT}}=\frac{\sum_{i=1}^{N}\sum_{j=1}^{M}{\left(BN_{{\mathrm{TF}\_S}_{i,j}}-BN_{\mathrm{TF}\_S\_\mathrm{MEAN}}\right)}^{4}}{N\cdot M\cdot BN_{\mathrm{TF}\_S\_\mathrm{SD}}{}^{4}}$
Coefficient of Variation	$BN_{\mathrm{TF}\_S\_\mathrm{CoV}}=\frac{BN_{\mathrm{TF}\_S\_\mathrm{SD}}}{BN_{\mathrm{TF}\_S\_\mathrm{MEAN}}}$
Spectral Entropy	$BN_{\mathrm{TF}\_S\_\mathrm{SE}}=\frac{1}{1-\alpha }\log_{2}{\sum_{i=1}^{N}\sum_{j=1}^{M}\left(\frac{BN_{{\mathrm{TF}\_S}_{i,j}}}{\sum_{i=1}^{N}\sum_{j=1}^{M}BN_{{\mathrm{TF}\_S}_{i,j}}}\right)}^{\alpha }$
Spectral Flatness	$BN_{\mathrm{TF}\_S\_\mathrm{SF}}=M\cdot N\frac{\prod_{i=1}^{N}{\prod_{j=1}^{M}\left|BN_{{\mathrm{TF}\_S}_{i,j}}\right|}^{\frac{1}{N\cdot M}}}{\sum_{i=1}^{N}\sum_{j=1}^{M}BN_{{\mathrm{TF}\_S}_{i,j}}}$
Concentration measure	$BN_{\mathrm{TF}\_S\_\mathrm{CM}}={\left(\sum_{i=1}^{N}\sum_{j=1}^{M}{\left|BN_{{\mathrm{TF}\_S}_{i,j}}\right|}^{\frac{1}{p}}\right)}^{p}$

^1^ Similarly same features set was calculated for real *BN*_TF_R_ and imaginary *BN*_TF_I_ part of *S*_BN_(*t*, *f*). In accordance, the type of the *TF* representation type from which a given feature is calculated is denoted by letter S, R or I in reference to the spectrogram, real or imaginary part.

## References

[1] Cullity B.D., Graham C.D. (2008). Domains and magnetization process. Introduction to Magnetic Materials.

[2] Hubert A., Schäfer R. (1998). Magnetic Domains—The Analysis of Magnetic Microstructures.

[3] Jiles D. (2015). Introduction to Magnetism and Magnetic Materials.

[4] Kleber X., Vincent A. (2004). On the role of residual internal stresses and dislocations on Barkhausen noise in plastically deformed steel. NDT E Int..

[5] Kikuchi H., Ara K., Kamada Y., Kobayashi S. (2011). Characteristics of barkhausen noise properties and histeresis loop on tensile stressed rolled steels. J. Magn..

[6] Lopato P., Psuj G., Herbko M., Maciusowicz M. (2016). Evaluation of stress in steel structures using electromagnetic methods based on utilization of microstrip antenna sensor and monitoring of AC magnetization process. Inf. Cntrl. Meas. Econ. Environ. Prot..

[7] Jiles D.C. (1990). Microstructure and stress dependence of the magnetic properties of steels. Rev Progress Quant. Nondestr. Eval..

[8] Chady T. (2002). Evaluation of stress loaded steel samples using GMR magnetic field sensor. IEEE Sens. J..

[9] Kwaśniewski J., Roskosz M., Witoś M., Molski S. (2018). Applications of magnetometric sensors based on amorphous materials in diagnostics of wire ropes. Arch. Min. Sci..

[10] Liu T., Kikuchi H., Ara K., Kamada Y., Takahashi S. (2006). Magnetomechanical effect of low carbon steel studied by two kinds of magnetic minor hysteresis loops. NDT E Int..

[11] Ding S., Tian G., Dobmann G., Wang P. (2017). Analysis of domain wall dynamics based on skewness of magnetic Barkhausen noise for applied stress determination. J. Magn. Magn. Mat..

[12] Psuj G. (2018). Multiple parameters fusion of electromagnetic nondestructive inspection data for evaluation of fatigue damage in steel elements. Int. J. Appl. Electromagn. Mech..

[13] Drehmer A., Gerhardt G.J.L., Missel F.P. (2013). Case depth in SAE 1020 steel using Barkhausen noise. Mat. Res..

[14] Sorsa A., Santa-Aho S., Vippola M., Lepisto T., Leiviska K. (2014). Utilization of frequency-domain information of Barkhausen noise signal in quantitative prediction of material properties. AIP Conference Proceedings.

[15] Kownacki C. (2008). Wavelet analysis of Barkhausen noise in reconstructing distributions of residual stress. Sol. St. Phen..

[16] Miesowicz K., Staszewski W.J., Korbiel T. (2016). Analysis of barkhausen noise using wavelet-based fractal signal processing for fatigue crack detection. Int. J. Fatig..

[17] Grijalba F.A.F., Padovese L.R. (2018). Non-destructive scanning for applied stress by the continuous magnetic Barkhausen noise method. J. Magn. Magn. Mat..

[18] Pérez-Benitez J.A., Padovese L.R., Capó-Sánchez J., Anglada-Rivera J. (2003). Investigation of the magnetic Barkhausen noise using elementary signals parameters in 1000 commercial steel. J. Magn. Magn. Mat..

[19] Deng Y., Li Z., Chen J., Qi X. (2018). The effect of the structure characteristic on magnetic Barkhausen noise in commercial steels. J. Magn. Magn. Mat..

[20] Makowska K., Kowalewski Z.L., Augustyniak B., Piotrowski L. (2014). Determination of mechanical properties of P91 steel by means of magnetic Barkhausen emission. J. Theor. Appl. Mech..

[21] Padovese L., Martin N., Millioz F. (2009). Time-frequency and time-scale analysis of Barkhausen noise signals. Proc. Inst. Mech. Eng..

[22] Psuj G., Maciusowicz M. Analysis of time-frequency representation of magnetic Barkhausen noise for the need of damage evaluation of steels elements. Proceedings of the 2018 International Interdisciplinary PhD Workshop (IIPhDW).

[23] Maass P., Teshke G., Willmann W., Wollmann G. (2000). Detection and classification of material attributes—A practical application of wavelet analysis. IEEE Trans. Sign. Process..

[24] Psuj G. (2015). Fusion of multiple parameters of magnetic testing results for damage assessment of loaded steel structures. Studies in Applied Electromagnetics and Mechanics: Electromagnetic Nondestructive Evaluation (XVI).

[25] Psuj G., Maciusowicz M., Chudzik P. Influence of measurement conditions on the magnetic Barkhausen noise properties. Proceedings of the 2018 International Interdisciplinary PhD Workshop (IIPhDW).

[26] Kypris O., Nlebedim I.C., Jiles D.C. (2014). A model for the Barkhausen frequency spectrum as a function of applied stress. J. Appl. Phys..

[27] Tomkowski R., Jonsson S., Lundin P., Nerman P. Penetration depth investigation of Barkhausen noise signal for case-hardened components. Proceedings of the 12th International Conference on Barkhasuen Noise and Micromagnetic Testing.

[28] Yamazaki T., Furuya Y., Nakao W. (2019). Experimental evaluation of domain wall dynamics by Barkhausen noise analysis in Fe_30_Co_70_ magnetostrictive alloy wire. J. Magn. Magn. Mat..

[29] Nahak B. (2017). Material charakterization using Barkhausen noise analysis technique—A review. Indian J. Sci. Tech..

[30] Luo X., Wang Y., Zhu B., Zhang Y., Zhang Y. (2015). Super-resolution spectral analysis and signal reconstruction of magnetic Barkhausen noise. NDT E Int..

[31] Kowalczyk J., Libera M., Jósko M. (2005). Evaluation of rolling bearings elements surface layer state changes by means of Barkhausen noise. J. Res. Appl. Agric. Eng..

[32] Capo Sanchez J., de Campos M.F., Padovese L.R. (2017). Comparison between different experimental set-ups for measuring the magientic barkhausen noise in a deformed 1050 steel. J. Nondes Eval..

[33] Dhar A., Atherton D.L. (1992). Influence of magnetizing parameters on magnetic barkhausen noise. IEEE Trans. Magn..

[34] Wang P., Gao Y., Yang Y., Tian G., Yao E., Wang H. (2013). Experimental studies and new feature extraction of mbn for stress measurement on rail tracks. IEEE Trans. Magn..

[35] Stefanita C.-G., Atherton D.L., Clapham L. (2000). Plastic versus elastic deformation effects on magnetic Barkhausen noise in steel. Acta Mater..

[36] Stewart D.M., Stevens K.J., Kaiser A.B. (2004). Magnetic Barkhausen noise analysis of stress in steel. Curr. Appl. Phys..

[37] Anglada-Rivera J., Padovese L.R., Capó-Sánchez J. (2001). Magnetic Barkhausen noise and hysteresis loop in commercial carbon steel: influence of applied tensile stress and grain size. J. Magn. Magn. Mater..

[38] Gathelier-Rothea C., Chicois J., Fougeres R., Fleischmann P. (1998). Characterization of pure iron and (130p.p.m.) carbon–iron binary alloy by Barkhausen noise measurements: Study of the influence of stress and microstructure. Acta Mater..

[39] Piotrowski L., Augustyniak B., Chmielewski M., Kowalewski Z. (2010). Multiparameter analysis of the Barkhausen noise signal and its application for the assessment of plastic deformation level in 13HMF grade steel. Meas. Sci. Technol..

[40] Bartosova I., Veternikova J., Slugen V. (2014). Study of candidate materials for new reactor system using positron annihilation spectroscopy and Barkhausen noise. Nuc. Eng. Des..

[41] Montinaro N., Cerniglia D., Pitarresi G. (2018). Evaluation of interlaminar delamination in titanium-graphite fibre metal laminates by infrared NDT techniques. NDT E Int..

[42] Cerniglia D., Montinaro N., Nigrelli V. (2008). Detection of disbonds in multi-layer structures by laser-based ultrasonic technique. J. Adhes..

[43] Bastiaans M.J. (1980). Gabor’s Expansion of a Signal into Gaussian Elementary Signals. Proc. IEEE.

[44] Lopato P., Herbko M. (2018). A circular microstrip antenna sensor for direction sensitive strain evaluation. Sensors.

[45] Wilczyński T.J., Gelman L., Kleczkowski P. Spectral features of the clarinet sound revealed by the set of stft-based parameters. Proceedings of the 18th World Converence on Nondestructive Testing.

[46] Tsai A., Luh J., Lin T. (2015). A novel STFT-ranking feature of multi-channel EMG for motion pattern recognition. Expert Syst. Appl..

[47] Paliwal K.K., Alsteris L.D. (2005). On the usefulness of STFT phase spectrum in human listening test. Speech Comm..

[48] Sandsten M. (2018). Time-Frequency Analysis of Time-Varying Signals and Non-Stationary Process, An Introduction.

[49] Nisar S., Khan O.U., Tariq M. (2016). An Efficient Adaptive Window Size Selection Method for Improving Spectrogram Visualization. Comput. Intell. Neurosci..

[50] Yin Q., Shen L., Lu M., Wang X., Liu Z. (2013). Selection of optimal window length using STFT for quantitative SNR analysis of LFM signal. J. Syst. Eng. Elect..

[51] Rymarczyk T., Kozłowski E., Kłosowski G. Object analysis using machine learning to solve inverse problem in electrical impedance tomography. Proceedings of the 2018 IEEE International Conference on Imaging Systems and Techniques (IST).

[52] Psuj G. (2018). Multi-sensor data integration using deep learning for characterization of defects in steel elements. Sensors.

[53] Liu W., Liu Z., Núñez A., Wang L., Liu K., Lyu Y., Wang H. (2018). Multi-objective performance evaluation of the detection of catenary support components using DCNNs. IFAC-PapersOnLine.

[54] Mao K., Lu D., Tan Z. (2018). A case study on attribute recognition of heated metal mark image using deep convolutional neural networks. Sensors.

[55] Kong X., Li J. (2018). Vision-based fatigue crack detection of steel structures using video feature tracking. Comp. Aid. Civ. Infrastruc. Eng..

[56] Boashash B., Ouelha S. (2018). Designing high-resolution time-frequency and time-scale distributions for the analysis and classification of non-stationary signals: a tutorial review with a comparison of features performance. Digit. Sig. Proc..

[57] Boashash B., Ouelha S. (2016). Automatic signal abnormality detection using time-frequency features and machine learning: A newborn EEG seizure case study. Know. Bas. Syst..

[58] Lerch A. (2012). Instantaneous features, and Intensity. An Introduction to Audio Content Analysis: Application in Signal Processing and Music Informatics.

[59] Lopato P., Chady T. (2013). Pulsed terahertz inspection of non-conducting sandwich composites. AIP Conference Proceedings.

[60] Stanković L. (2001). A measure of some time–frequency distributions concentration. Sig. Proc..

[61] Sorsa A., Leiviskä K., Santa-Aho S., Lepistö T. (2012). Quantitative prediction of residual stress and hardness in case-hardened steel based on the Barkhausen noise measurement. NDT E Int..

